# P-1624. COVID-19 Vaccination Uptake following COVID-19 Infection in Unvaccinated and Previously Vaccinated Adults

**DOI:** 10.1093/ofid/ofaf695.1800

**Published:** 2026-01-11

**Authors:** Lakshmi Chauhan, Samantha Roberts, Lindsey Fish, Nichole Carlson, Adit A Ginde

**Affiliations:** University of Colorado, Aurora, CO; University of Colorado, Aurora, CO; Denver Health, Denver, Colorado; University of Colorado, Aurora, CO; University of Colorado, Aurora, CO

## Abstract

**Background:**

Uptake of COVID-19 vaccinations has declined significantly despite decreased immune protection from previous COVID-19 vaccines. In this study, we evaluated the factors influencing vaccine uptake and timing of vaccination following a COVID-19 diagnosis.

**Table 1:**
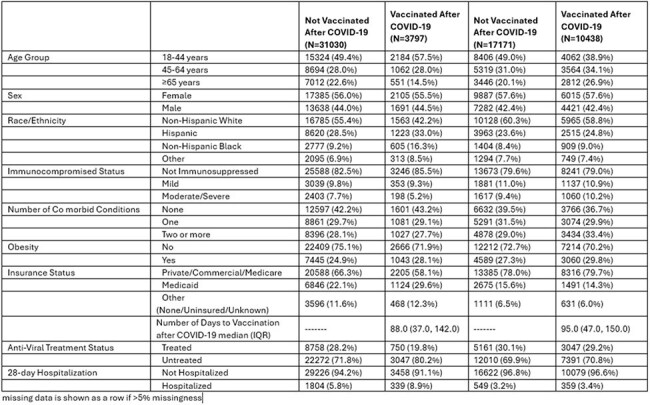
Demographic and Co-morbid Conditions by Vaccination Status

**Table 2:**
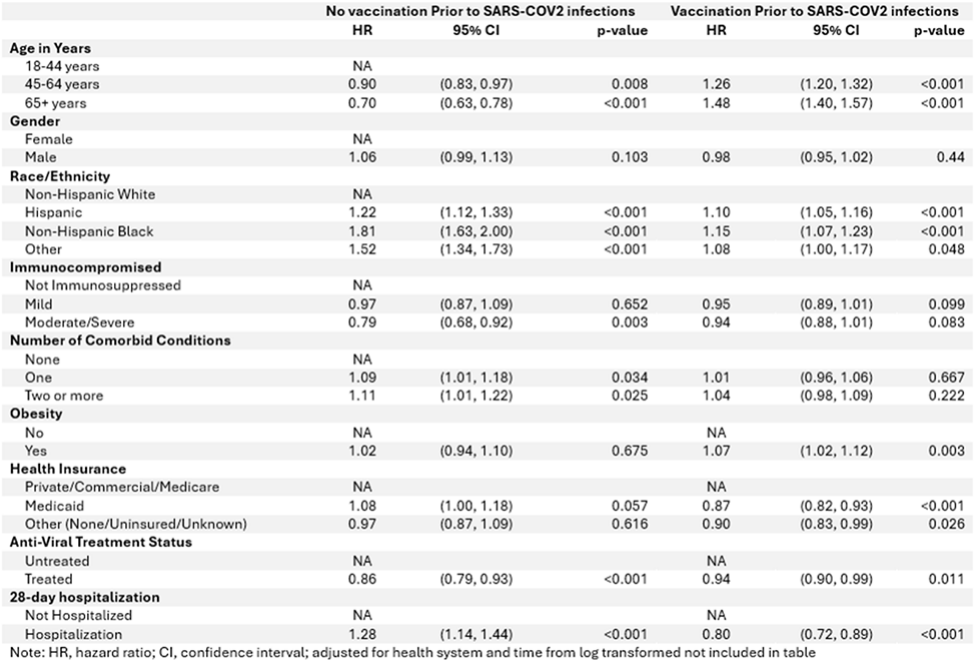
Adjusted Cox PH models for Vaccination after COVID-19

**Methods:**

We conducted a multicenter observational cohort study at University of Colorado and Denver Health; all patients diagnosed with SARS-CoV-2 infection between May 1, 2021, and January 4, 2023, were included. Primary outcome was the minimum number of days to vaccination or last day of follow-up. We performed multivariable Cox proportional hazards model to assess the associations between time to vaccination after SARS-CoV-2 infection and anti-viral treatment, 28-day hospitalization for COVID-19, age, race/ethnicity, gender, co-morbid conditions, immunocompromised status, and insurance.

**Figure 1:**
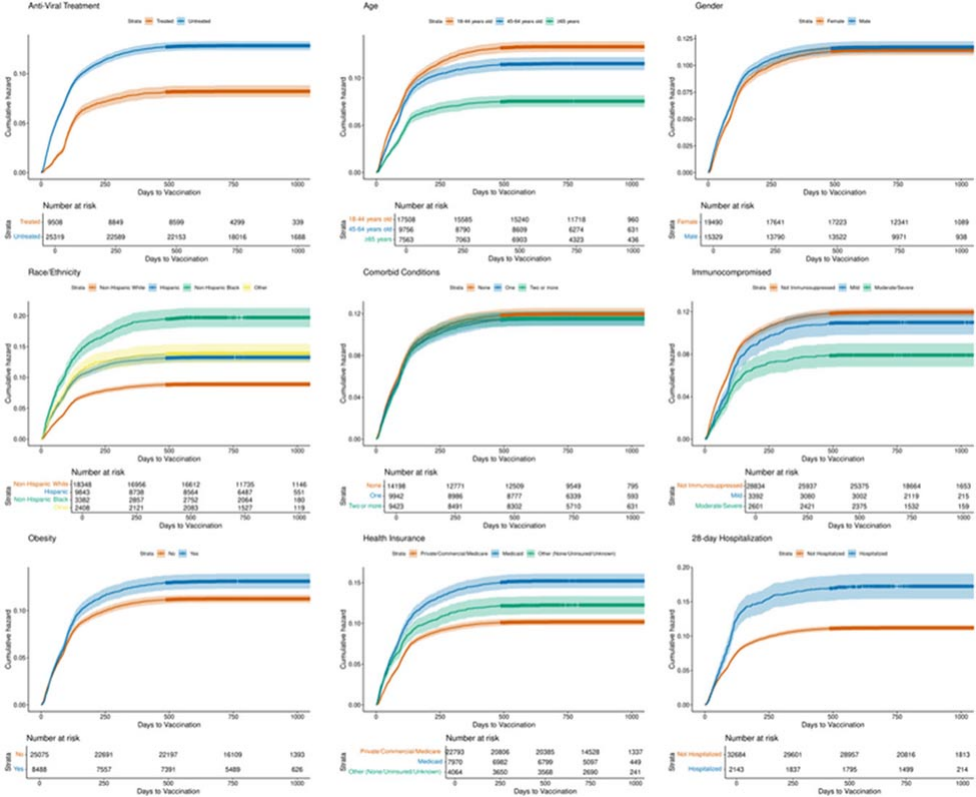
Kaplan Meier Curve of Day of Vaccination in Unvaccinated before COVID-19 Population

**Figure 2:**
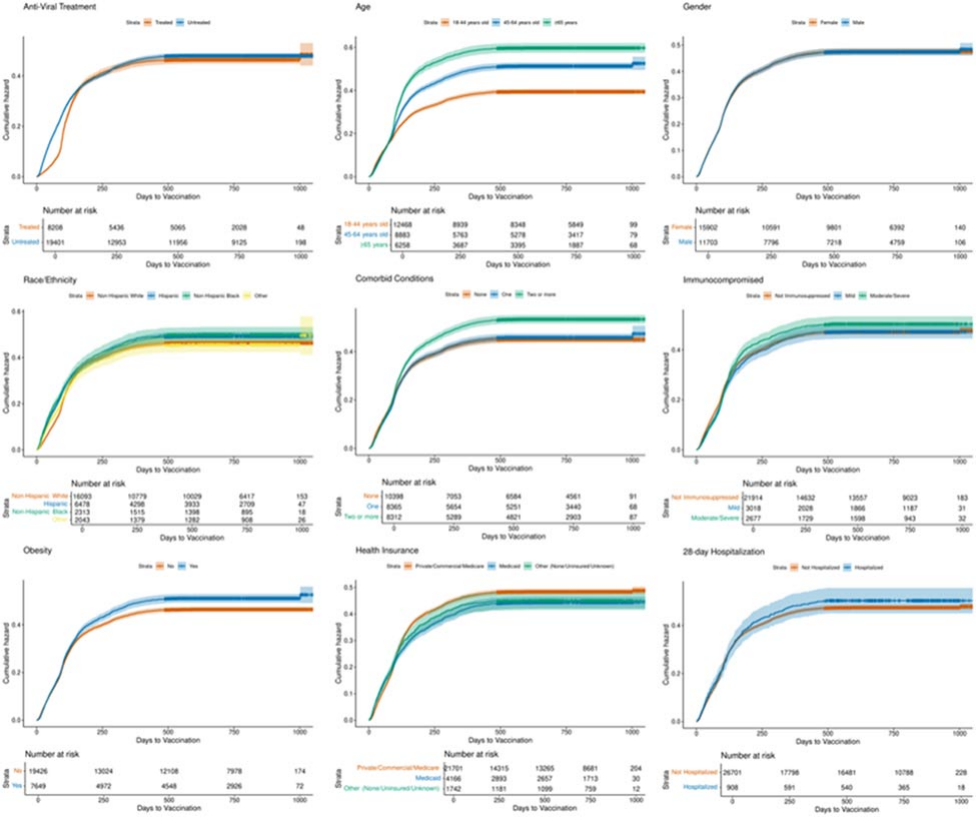
Kaplan Meier Curve of Day of Vaccination in Vaccinated before COVID-19 Population

**Results:**

96,395 patients were diagnosed with COVID-19, of which 61,568 (63%) had at least one vaccination prior to COVID-19 diagnosis. Of the 34,827 unvaccinated patients, only 3797 (12%) received vaccination after COVID-19 diagnosis with a median of 88 days to vaccination. Black (aHR 1.81, p< 0.001), Hispanic (aHR 1.22, p< 0.001), older adults (patients with co-morbid conditions (aHR 1.11, p= 0.025), and requiring hospitalization (aHR 1.28, p < 0.001) were more likely to receive their first vaccination after COVID-19. Older adults (aHR 0.7, p < 0.001), severely immunocompromised (aHR- 0.79, p 0.003) and those with antiviral use (aHR 0.86, p< 0.001) were less likely to receive vaccine. Of 27609 who received 1 or 2 vaccines, 10438 (38%) received vaccination after COVID-19 diagnosis. Older adults (aHR 1.48, p < 0.001), Hispanic (aHR 1.1, p < 0.001), and Black (aHR 1.15, p< 0.001) were more likely to receive vaccine after infection.

**Conclusion:**

Over a third of people presenting with COVID-19 diagnosis were unvaccinated. Previously unvaccinated individuals had low vaccine uptake even after an episode of COVID-19 with surprisingly lower uptake in older and immunocompromised adults. Previously vaccinated adults were more likely to receive vaccination after infection. Identifying groups at risk of severe infection with lower propensity to receive vaccination will enable targeted interventions to increase vaccine uptake.

**Disclosures:**

All Authors: No reported disclosures

